# Approximating complex 3D curves using origami spring structures

**DOI:** 10.1038/s44172-023-00149-1

**Published:** 2023-12-12

**Authors:** Zuolin Liu, Zian Zhang, Hongbin Fang

**Affiliations:** Institute of AI and Robotics, State Key Laboratory of Medical Neurobiology, Engineering Research Center of AI & Robotics, Fudan University, Shanghai, 200433 China

**Keywords:** Mechanical engineering, Design, synthesis and processing

## Abstract

Origami provides a versatile platform for creating intricate three-dimensional (3D) reconfigurable structures through folding techniques. However, the applications of origami patterns are restricted due to limited deformation modes and complex actuation. Here we explore origami spring structures as a solution to address these limitations by approximating complex 3D curves with an underactuated scheme. By doing so, we showcase the reconfigurability and versatility of origami springs while tackling control complexity. Through the introduction of virtual creases, we simplify non-rigid deformations and enable accurate descriptions of their 3D configurations. Furthermore, we develop inverse kinematics optimization algorithms to determine optimal configurations closely approximating given 3D curves with full actuation and underactuated situations. Experimental realization of various 3D curves demonstrates the feasibility and effectiveness of our proposed approach. This research could find practical utility in soft robotics, flexible mechanisms, and deployable structures.

## Abstract (web summary)

Liu, Zhang and Fang propose and experimentally demonstrate an optimization method for shape morphing of origami spring structures to approximate complex 3D curves by introducing virtual creases.

## Introduction

Origami, an ancient art of paper folding, has gained substantial attention across scientific and engineering disciplines due to its unique properties. Its ability to transform flat sheets of paper into intricate three-dimensional structures through folding techniques has provided a versatile platform for innovation. Researchers have recognized the immense potential of origami in the design and fabrication of structures capable of adaptation, morphing, and reconfiguration, resulting in notable advancements in fields like aerospace engineering^1^, robotics^2^, and material science^3^.

Extensive research has been dedicated to exploring various origami patterns in the domain of reconfigurable structures. Notable patterns such as Miura pattern^4^, Kresling pattern^5^, Origami ball pattern^6^, and Yoshimura pattern^7^ have been extensively investigated. Each pattern possesses distinct folding modes that give rise to specific deformation characteristics. For example, the Miura pattern enables axial expansion and contraction, making it well-suited for applications requiring compact storage and transportation^8^. The Origami ball pattern facilitates radial expansion, allowing for transformations into spherical configurations^9^. The Kresling pattern offers combined twisting and stretching deformations, providing added flexibility in achieving desired configurations^10^. Similarly, the Yoshimura pattern exhibits bending and compression deformation, enhancing the versatility of reconfigurable folded structures^11^. Despite their notable contributions, these patterns often have limitations in terms of their deformation capabilities, which are specific to certain modes.

To expand the range of deformation modes in origami structures, researchers have proposed variant approaches, including modifying geometry parameters and introducing additional folding creases to established patterns. These variant designs allow for great versatility and complexity. For example, the cylindrical Kresling origami incorporates free-form quadrilateral unit cells, resulting in diverse conical folded configurations^12^. Studies have also explored the addition of extra valley creases in the Kresling pattern, enabling multiple deformation modes of the inflated structure^13^. To achieve more notable shape changes in the Miura origami, a variation strategy^14^ or a digital design technique^15^ have been introduced during the stacking process. Another approach involves incorporating stretchable skins into conventional folding patterns, resulting in dual-mode morphing and enhanced shape changes^16^. Moreover, researchers have developed entirely new origami designs to unlock new possibilities for reconfigurable structures^17–19^. These innovative approaches open up new avenues for the exploration and advancement of reconfigurable origami structures.

While progress has been made in expanding the deformation modes of reconfigurable origami structures, it is important to acknowledge that different folding patterns still have limitations in their specialization of deformation modes. However, origami springs, formed by folding two paper strips against each other, offer a wide range of deformation capabilities, including stretching, twisting, bending, and more. The versatility of origami spring structures introduces new opportunities for exploring reconfiguration and achieving complex and multifaceted deformations in origami structures. Despite their potential, current research primarily focuses on axial stretch-twist coupling^20^, with a limited investigation into off-axial deformation. This gap in understanding the full potential of origami spring structures motivates further exploration into comprehending and investigating their complete spectrum of capabilities, particularly in terms of 3D reconfigurability. This research, therefore, aims to bridge the gap and uncover the intricate multi-model deformation behavior exhibited by origami springs.

Although the desire to achieve complex 3D shape customization in origami structures has long been pursued^21,22^, it is important to acknowledge the associated challenges in terms of actuation and control. As the number of folding degrees of freedom increases, the need for a greater number of actuators to achieve reconfigurable shapes becomes apparent. This increased demand for control presents a huge obstacle. Traditional motor actuation, for example, requires numerous actuators to fold a modular robotic arm into a 3D configuration^23^. Alternatively, smart actuators such as pneumatic drives^2^, thermal drives^24^, and magnetic drives^25^ reduce the number of actuators but often sacrifice actuation simplicity and accuracy. Some researchers have explored utilizing the dynamics of origami structures for reconfiguration, allowing a single actuator to switch the structure’s configuration^26^. Note that the above approaches are sensitive to external loading and require high actuation accuracy. The challenge lies in reconfiguring a flexible origami structure to achieve complex 3D shapes while using limited actuators, maintaining simplicity in actuation, and ensuring the accuracy of configuration. To address this challenge, there is a need to explore underactuated control techniques. Underactuation refers to a system where the number of control inputs is fewer than the degrees of freedom, enabling simplified control strategies by leveraging the compliant elements within the structure. Despite the potential benefits, the application of underactuated control techniques for achieving complex shape reconfiguration of origami structures remains relatively unexplored.

In this study, we present a comprehensive investigation into the potential of origami spring structures for approximating complex 3D curves and achieving intricate configurations with a limited number of actuators. Our research contributes to the advancement of reconfigurable origami structures and soft robotics by showcasing the versatility and adaptability of origami springs while minimizing control complexity. To accomplish this, we simplify the non-rigid deformation of origami spring structures into rigid folding by introducing virtual creases. Through rotation and translation transformations, we establish the spatial kinematics of origami spring structures, providing an accurate description of their spatial configuration. In addition, we develop an inverse kinematics optimization algorithm that determines the optimal configuration of the origami spring structure to closely approximate a given 3D curve, such as an ‘S’-shape, ‘J’-shape, spiral-shape, and other complicated 3D shapes. To address the underactuated scenario, we further introduce additional constraints to the inverse kinematics optimization algorithm, enabling effective shape customization with limited actuators. To validate our proposed approach, we fabricate physical prototypes of the origami spring structures and measure their deformation behavior, comparing them with the expected results based on our proposed method. The experimental verifications provide evidence of the feasibility and effectiveness of the proposed optimization-based approach. By enhancing our understanding of origami spring structures and proposing control techniques, we open up possibilities in reconfigurable origami structures, soft robots, flexible mechanisms, and morphing structures, enabling the development of more adaptable and efficient solutions.

## Results

### Geometry and kinematics of origami spring structures

**Fig. 1:**
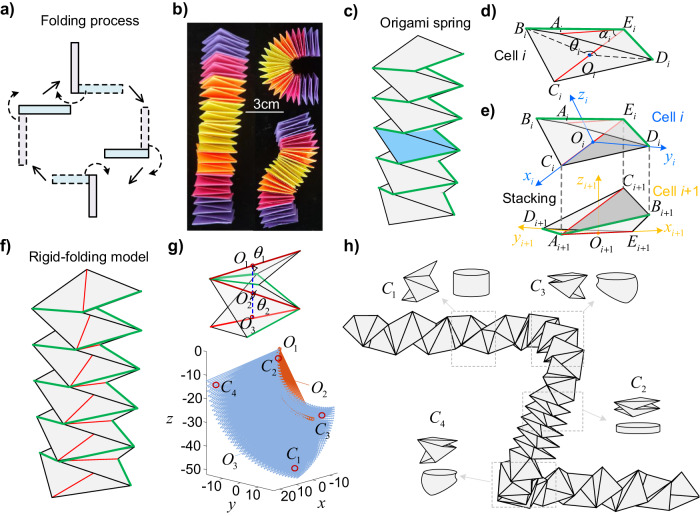
Kinematics of the origami spring structure. **a** Illustration of the folding process of the origami spring structure. **b** Photos showing the multi-mode deformation. **c** Illustration of a non-rigid-foldable origami spring structure, where the green lines represent the folding creases, and a representative unit cell is marked in blue. **d** Simplification of a non-rigid-foldable cell into a rigid-foldable origami by introducing virtual creases (depicted by red lines). **e** The rotation and translation transformations of the stacking. The shared facet is denoted in gray. **f** Illustration of the rigid foldable origami spring structure. **g** Schematic diagram illustrating two cells (top panel) and their reachable workspace (bottom panel). Four qualitatively distinct deformation modes are labeled as ${C}_{1}$, ${C}_{2}$, ${C}_{3}$ and ${C}_{4}$, which correspond to axial stretching, axial compressing, bending with ${\theta }_{1} < {\theta }_{2}$, and bending with ${\theta }_{1} > {\theta }_{2}$, respectively. **h** 3D shape of an origami spring structure achieved by combining the four distinct deformation modes of a cell.

The origami spring is one of the simplest origami structures that can be constructed by alternatively folding two perpendicularly arranged rectangular paper strips of the same size (Fig. 1a). Generally, origami springs exhibit a similar resting state and deformation pattern along the axial direction as linear springs (left panel in Fig. 1b); however, when external forces are applied in off-axis directions, various deformation modes can be observed (right panels in Fig. 1b). To thoroughly investigate the deformability of origami spring structures and eventually achieve precise spatial shape programmability, a detailed analysis of the geometry and kinematics of the origami structure is conducted.

Note that the origami spring structure is periodic (Fig. 1c), i.e., it consists of repeated arrangements of a basic cell. Therefore, the geometry of the *i* ^th^ single cell (highlighted in blue in Fig. 1c) is analyzed in this paper. The cell consists of two square facets, and the length of their creases is denoted as $\sqrt{2}a$. During deformation, the square facets undergo an out-of-plane bending mode. To simplify the analysis, virtual creases (red lines in Fig. 1d) are introduced along the diagonals of the facets. This allows for quantifying the non-rigid deformation through the rotation of the triangular facets. It is worth noting that the deformation behaviors of the origami structure after introducing into virtual creases are very close to that of the non-rigid-foldable origami spring structures, thus facilitating the kinematic analysis. This also allows us to customize spatial curve design through inverse kinematics optimization in subsequent studies. Therefore, the following theoretical and experimental analyses will be centered around the rigid-foldable origami spring structures incorporating virtual creases. This approach promises to provide a more convenient solution for understanding and manipulating the deformation of non-rigid-foldable origami structures, facilitating the advancement of both theory and practical applications.

The simplified unit cell is a one-degree-of-freedom system. Its folding can be uniquely characterized by folding angle ${\theta }_{i}$ (i.e., $\angle {D}_{i}{O}_{i}{B}_{i}$, ${O}_{i}$ lies in the middle of ${C}_{i}{E}_{i}$). For an origami spring structure with *n* unit cells, adjacent cells share a triangular plane (Fig. 1e, f), so the kinematics can be determined by the rotation and translation transformation. A detailed derivation of the kinematics is given in the Methods section.

Utilizing the derived kinematics, we can explore the reachable workspace of the origami spring structure. Figure 1g showcases the reachable workspace of an origami spring structure comprising two cells. Within this accessible space, four qualitatively distinct deformation modes are identified and labeled as ${C}_{1}$, ${C}_{2}$, ${C}_{3}$ and ${C}_{4}$, ${C}_{1}$ representing axial stretching, axial compressing, bending with ${\theta }_{1} < {\theta }_{2}$, and bending with ${\theta }_{1} > {\theta }_{2}$, respectively (Fig. 1h). These fundamental deformation modes can be combined to reconfigure origami spring structures with multiple cells into intricate 3D spatial shapes. An example of such a configuration is depicted in Fig. 1h. This demonstrates the capability of the origami spring structure to achieve complex and versatile transformations.

It is noted that while a single cell has only one degree of freedom, an origami spring structure composed of multiple cells has multiple degrees of freedom. Effectively tailoring the angles of constituent cells to approximate intricate spatial curves is a challenging task, especially given the requirement to minimize the number of actuators to simplify the control complexity, and this is one of the issues we will address subsequently.

### Approximating 3D curves with full actuation: an optimization-based approach

**Fig. 2:**
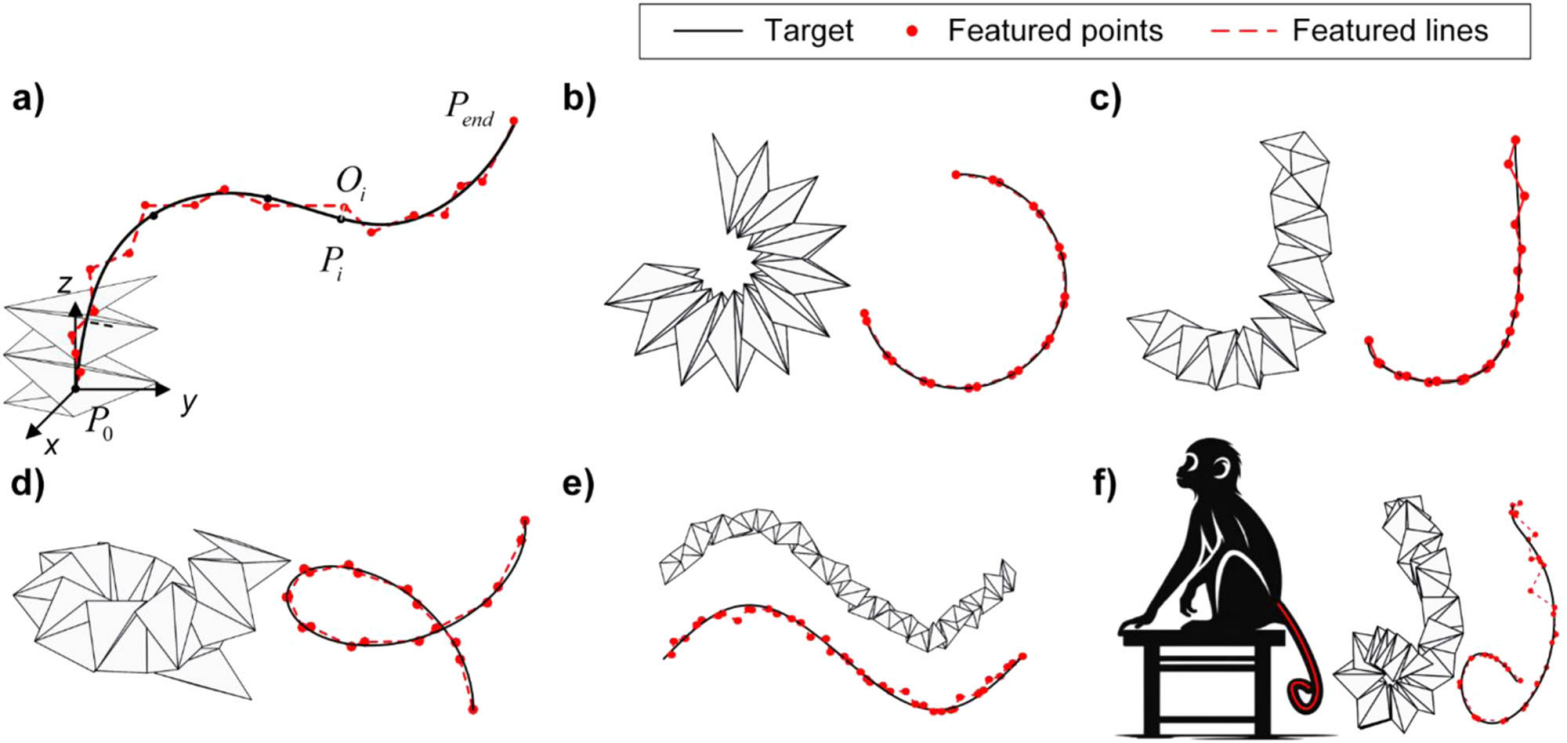
Shape customization of the origami spring structure for approximating diverse 3D curves. This highlight its rich reconfigurability and effectiveness of the optimization approach. The black and red lines denote the target curve and the featured lines on the origami spring structures. ${O}_{i}$ is the featured point of the origami spring structure, while ${P}_{i}$ refers to the point on the 3D curve closest to ${O}_{i}$. The coordinate origin is located at the featured point of the first cell. **a** Illustration of the target-oriented shape-reconfiguration of the origami spring structure. Shape customization of the origami spring structure for approximating **b** a ‘C’-shaped curve, **c** a ‘J’-shaped curve, **d** a spiral-shaped curve, **e** a sinusoidal-shaped curve, and **f** a monkey tail-shaped curve.

Given the rich reconfigurability of origami spring structures, they have the potential to approximate a variety of 3D curves. In this section, we elaborate on an optimization approach that is capable of accurately tuning the actuation angles of the constituent cells of origami spring structures, facilitating the approximation of various target curves. Since origami spring structures usually have a large number of cells and thus a high aspect ratio, they can be approximated as one-dimensional structures. To simplify the representation of the 3D shape of the origami spring structure, we introduce the concept of a center axis, which represents the characteristics of the origami spring structure along the length direction while disregarding its cross-sectional shape. This center axis is defined by points ${O}_{i}$, ($i=0,1,\cdots ,n+1$), where $n+2$ represent the total number of points. The aim is to approximate a target 3D curve, indicated by the black line in Fig. 2a. To achieve this approximation, we strive to minimize the distance between the featured points ${O}_{i}$ and the 3D curve. The distances can be measured by drawing perpendicular lines from the featured points to the curve, with the points of intersection denoted as ${P}_{i}$, ($i=0,1,\cdots ,n+1$). Consequently, the overall distance is determined by evaluating the distances between the featured points and their corresponding feet of perpendicular on the 3D curve. It is important to note that the first and last points of the origami spring are fixed on the target 3D curve. Therefore, the overall distance yields $S={s}_{1}+{s}_{2}\,+\cdots {s}_{n}$, ($\,{s}_{i}=\Vert {O}_{i}{P}_{i}\Vert \,$). The spatial configuration of the origami spring structure can therefore be obtained by minimizing the overall distance, see the detailed description of the optimization in the Methods section. The optimization process can be conducted by classical methods such as Gauss-Newton, stochastic gradient descent, Particle Swarm Algorithm, or Genetic Algorithm. Here we use the *fmincon* optimizer in Matlab for simplicity, which can successfully find the optimal solution. The optimization efficiency of these algorithms is not in the focus of this paper. To showcase the effectiveness of this approach, we first employ three distinct 3D curves with parametric equation expressions as target shapes: a ‘C’-shaped curve, a ‘J’-shaped curve, and a spiral-shaped curve. The parametric equations representing these curves are provided in Supplementary Notes.

Utilizing the proposed optimization method, we are able to illustrate the customized shape of the origami spring structure in Fig. 2b–d. It is evident that the feature points of the structure closely align with the target 3D curves, indicating a successful approximation. Noticing that S carries the dimension of length, it provides a quantitative expression for curve fitting error but remains scale-dependent. To eliminate the influence of scale, we introduce the relative error, denoted as $e=S/R\,$ ($R\,$ is the total arc length of the target curve). This adjustment allows for a scale-independent assessment of fitting accuracy. The resulting relative errors of the three approximations are all found to be less than 0.66% (see detailed results in Supplementary Table 4). This signifies a high level of consistency between the customized shape of the origami spring structure and the target 3D curves, demonstrating the effectiveness of the proposed optimization approach in achieving accurate shape customization of the origami spring structure.

In addition to simple 3D curves with explicit parametric equation expressions, we explore the customization of more complex curves, such as a sinusoidal 3D curve with a direct equation expression and even a monkey tail without an explicit equation expression. The approach involves transforming or fitting these 3D curves into parametric equations, which are then used in conjunction with the proposed method. Remarkably, the proposed method remains efficient in customizing the configuration of the origami spring structure, even for these more challenging curves. Figure 2e, f illustrates the results, showcasing the customized shapes of the origami spring structure that closely approximates the target curves. The relative error is calculated to be 0.60% and 1.81%, respectively, which demonstrates the versatility and power of the proposed method in achieving accurate shape customization, even when dealing with complex 3D curves that may not have explicit equation expressions. By transforming or fitting these curves into parametric equations, the origami spring structure can still be effectively reconfigured to match the desired shape, showcasing the adaptability and efficiency of the approach.

**Fig. 3:**
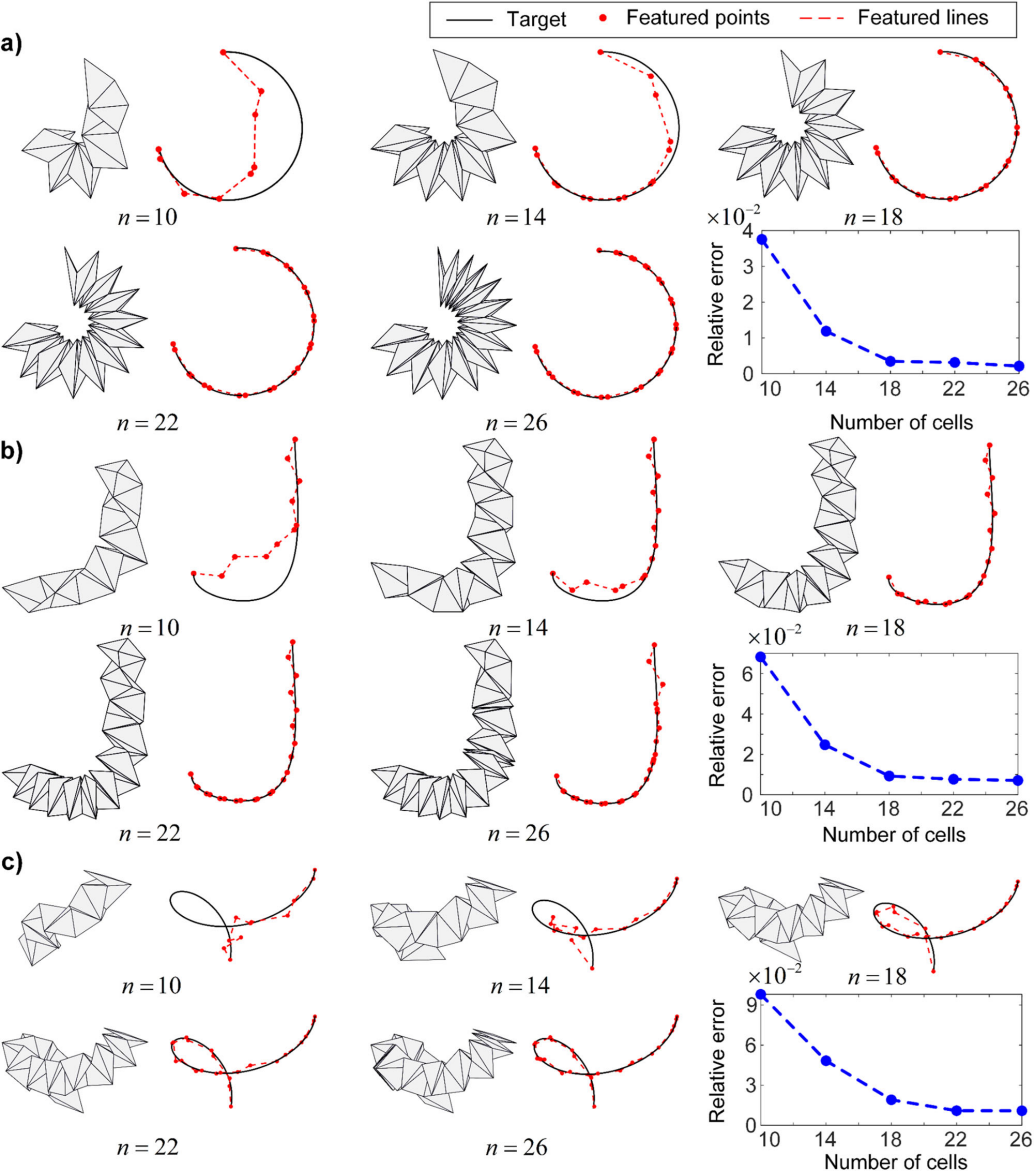
Effects of the number of constituent cells on the approximation of various 3D curves. $n$ refers to the number of constituent cells. Approximation results for **a** a ‘C’-shaped curve, **b** a ‘J’-shaped curve, and **c** a spiral-shaped curve. The number of constituent cells ranges from 10 to 26, and the corresponding approximating error is depicted in the bottom right panel in each subplot.

The number of constituent cells plays a crucial role in determining the reconfigurability of the origami spring structure. Intuitively, a larger number of cells allows for greater flexibility in customizing the shape to approximate various 3D curves. However, this increased flexibility comes at the cost of structural complexity. We reconsider the same 3D curves from Fig. 2b–d but with different numbers of cells. It is observed that with a small number of cells, the approximation of the three curves is poor (Fig. 3). However, as more cells are incorporated into the structure, the shape reconfigurability is greatly enhanced, resulting in decreased relative errors. This improvement becomes less evident as more cells are added. Eventually, when a sufficient number of cells is used, the error reduction becomes marginal. This suggests that an optimal balance can be achieved between the structural complexity and the approximating capability of the origami spring. By carefully selecting the number of constituent cells, it is possible to optimize the design of the origami spring, achieving a trade-off between the structural simplicity and the accuracy of the approximation.

### Approximating 3D curves with underactuated origami spring: an optimization-based approach

In practical applications, controlling multiple independent origami spring cells to achieve a desired target configuration can be challenging and resource-intensive, requiring a large number of actuators. This can result in increased fabrication and control costs. Therefore, we explore the possibility of achieving similar shape reconfigurability with fewer independent actuators, aiming to strike a balance between the achievable shape complexity and the control complexity, making it more practical and cost-effective in real-world applications.

In the scenario where there are $m$ ($m\le n$) independent actuators, the folding angles of the actuated unit cells are denoted as control angles ${\gamma }_{j}$ ($j=1,2,\cdots ,m$). Theoretically, each cell ${\theta }_{i}$ ($j=1,2,\cdots ,n$) can take one of the control angles. Particularly, when $m=n$, the control angle and the folding angle correspond to each other. Consequently, there can be millions of different combinations, resulting in a huge computation burden. To alleviate this computational challenge, we introduce the Lagrange multiplier method by minimizing the modified target $S+\lambda \mathop{\sum }\nolimits_{i=1}^{n}{({\theta }_{i}-{\gamma }_{1})}^{2}{({\theta }_{i}-{\gamma }_{2})}^{2}\cdots {({\theta }_{i}-{\gamma }_{m})}^{2}$, where $\lambda$ is the Lagrange multiplier which takes a large number in the optimization. Therefore, the folding angle ${\theta }_{i}$ would automatically approximate one of the control angles in order to minimize the overall loss. This process aims to find an initial approximation of the folding angles, which is defined as the first optimization. After the first optimization, although the folding angles are very close to the control angles, there may still be small differences between them. To further address this discrepancy, we proceed with a second optimization step. In this step, we directly set the close folding angles to be equal to the corresponding control angles. By doing so, we reduce the number of independent variables to be optimized. As a result, in the second optimization, there are only $m$ independent control angles that need to be optimized. The remaining folding angles have already been set to their corresponding control angles, eliminating the need for further adjustment. Details of the optimization method are presented in the Methods section. By conducting these two optimization steps, we are able to effectively approximate the desired shape while reducing the number of independent variables, which helps to streamline the optimization process and reduce computational complexity.

**Fig. 4:**
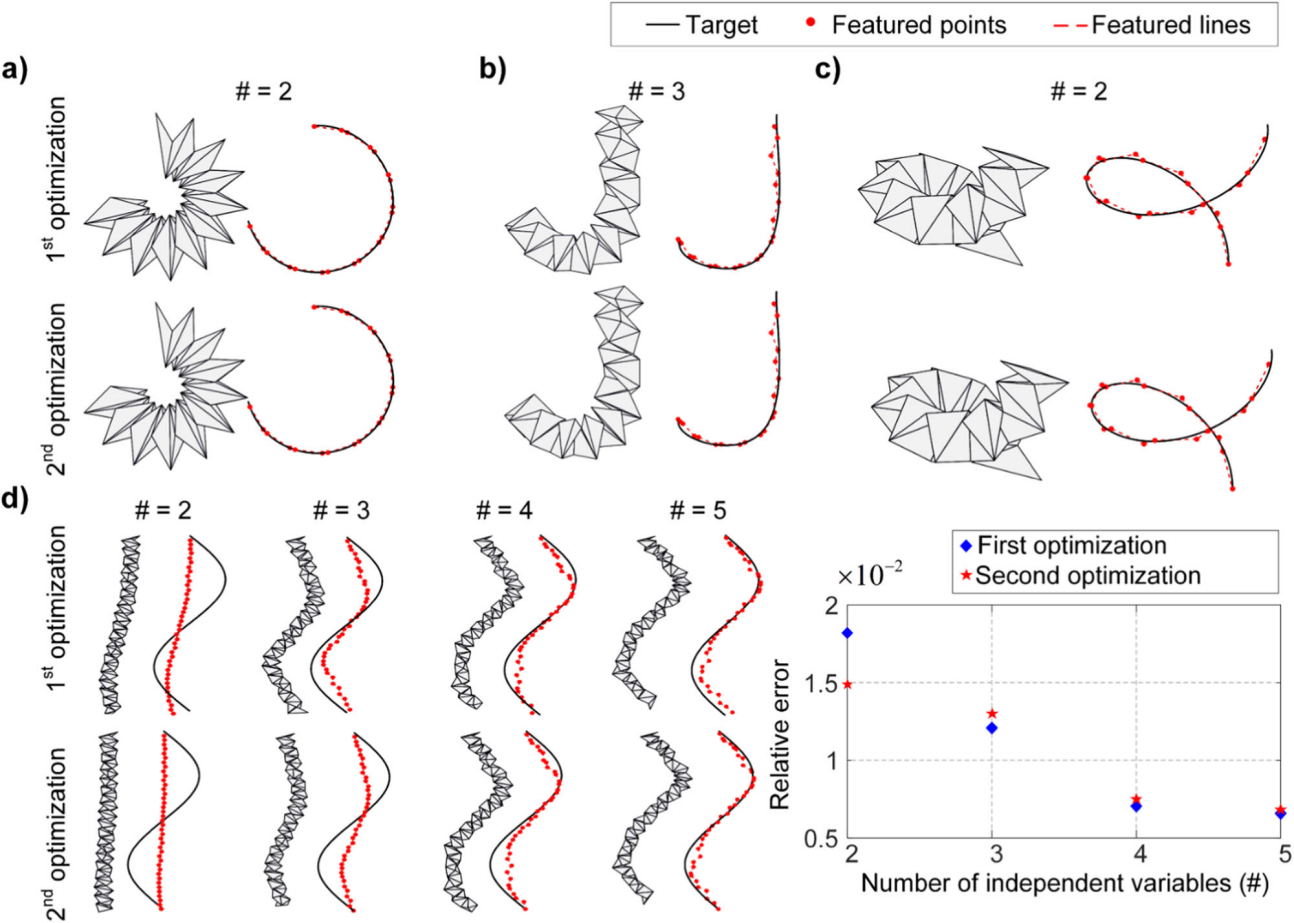
Shape customization of the origami spring structure for various 3D curves with limited actuators after first and second optimization. **a** Approximating a ‘C’-shaped curve with 2 actuators, **b** approximating a ‘J’-shaped curve with 3 actuators, **c** approximating a spiral-shaped curve with 2 actuators. **d** Approximating a sinusoidal-shaped curve with different numbers of actuators. The effects of the number of actuators for approximation are shown in the right panel.

To assess the efficiency of the proposed optimization approach, we apply it to approximate the aforementioned three curves using limited independent actuators. For ‘C’ and spiral-shaped curves, the independent actuators are two, while for the ‘J’-shaped curve, the independent actuators are set as three. While the spiral-shaped curves may appear visually complex, they share the same deformation mode as the ‘C’ curve, requiring the contraction of unit cells on one side. The primary difference lies in the degree of contraction, leading to the use of two actuators for both shapes. In contrast, the deformation mode for the ‘J’-shaped curve is more complex, where the upper section stays straight while the lower section forms a ‘C’ shape, which requires three actuators to realize. After the first optimization step, the resulting configuration of the origami spring structure exhibits a favorable agreement with the target curves (top panels in Fig. 4a–c), yielding a relative error of 0.32%, 0.91%, and 1.63%, respectively. The optimized folding angles are presented in Supplementary Tables 1–3. Remarkably, after the second optimization, where cells with similar angles are made equal to the corresponding control angles, the configurations of the origami spring structures remain very close to the given 3D curves (bottom panels in Fig. 4a–c). This observation highlights the remarkable ability of the underactuated origami spring structure to approximate various 3D curves.

It is noteworthy that the results for the ‘C’ and the spiral-shaped curves exhibit similarities. Specifically, both of them require two independent actuators and share the same arrangement of actuators. The only variation lies in the values of the actuation angles (Supplementary Table 1 and Supplementary Table 3). Through a systematic exploration of potential actuation angles, this deterministic structure demonstrates superior reconfigurability, encompassing ‘C’-shaped and spiral-shaped curves, as well as various other intricate configurations (see details in Supplementary Discussion 1). This observation underscores that the same arrangement of drivers can realize multiple distinct spatial configurations, thereby affirming the rich reconfigurability inherent in origami spring structures, even when they are underactuated.

When dealing with more complicated 3D curves, such as the sinusoidal curve shown in Fig. 4d, it becomes evident that a structure with only 2 actuators is insufficient for accurate approximation (left panel of Fig. 4d). In such cases, more actuators need to be incorporated to enhance the reconfigurability of the origami spring structure and improve the approximation accuracy. By increasing the number of independent actuators, a better fit to the target curve can be achieved. In the extreme case, if there are enough independent actuators to match the number of unit cells, the problem degenerates to full actuation (Fig. 2e), which notably improves the approximation. It is important to note that the computed outputs for the number of independent actuators and their control angles are not unique. Given a target curve, there can be multiple optimization results, all of them corresponding to good fitting accuracy, but the optimized number of actuators and the actuation angles are different. In practical applications, it is not always feasible or cost-effective to incorporate a large number of independent actuators. Therefore, it is important to consider the intermediate situation where the number of actuators is limited. Figure 4d provides insight into the relationship between the fitting error and the number of independent actuators. As expected, the fitting error decreases as the number of independent actuators increases. This suggests that a greater number of actuators enhances the reconfigurability of the origami spring structure, leading to an improved approximation of the 3D curve. However, it is important to note that the rate of improvement in fitting error diminishes as more independent actuators are introduced. This indicates that beyond a certain threshold, the performance improvement becomes less evident. The conflict between actuation complexity and reconfigurability can be balanced to achieve a better fit of 3D curves according to specific requirements. Designers should carefully weigh the number of independent actuators against the desired reconfigurability. To effectively approximate a 3D curve, the configuration needs to be optimized according to given constraints, taking into account factors such as manufacturing cost, control complexity, and target accuracy. In practice, we adopt a pragmatic strategy, starting with a single actuator and gradually increasing the number. We evaluate the decrease in fitting error resulting from each additional actuator, comparing it to a predefined threshold (e.g., 10% for curves in Fig. 4). If the reduction falls below this threshold, we consider the marginal benefit of adding a new actuator negligible and refrain from further increments. Since our goal is to reconfigure the origami spring structure to a specified spatial curve with as few actuators as possible, this incremental approach strikes a balance between approximation accuracy and actuation cost while minimizing the computational burden.

### Experiments

**Fig. 5:**
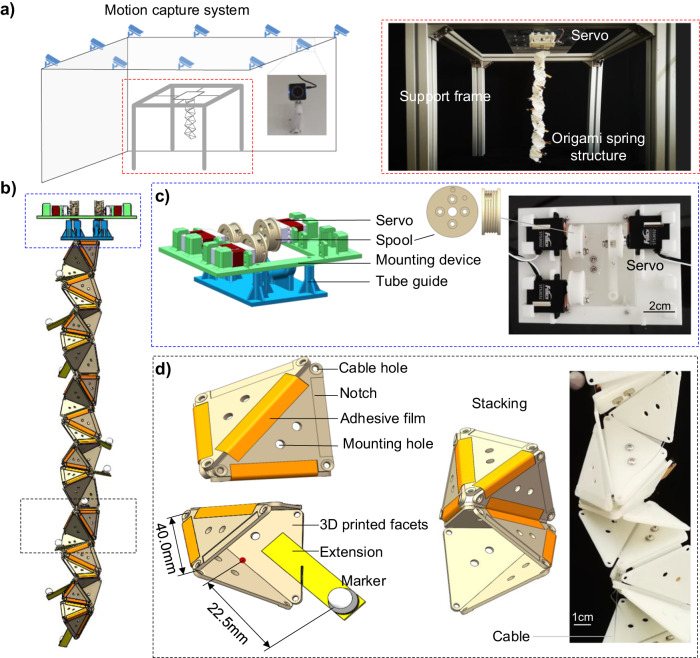
Fabrication and experimental setup. **a** Schematic diagram of the overall setup and photo of the experimental platform. **b** CAD design of the origami spring structure and the control system. **c** Detailed CAD design and photo of the control system. **d** Detailed diagram and photo of the origami spring structure.

The primary objective of the experiment is to verify the effectiveness of the optimization approach in approximating 3D curves, particularly in scenarios where limited actuators are employed. The experimental setup comprises three key components: the origami spring structure, the control system, and the motion acquisition system (as depicted in Fig. 5). These components work together to facilitate the experimental process and data collection. For motion acquisition, a commercial Vicon optical motion capture system is employed. This system consists of 10 high-speed optical cameras that precisely measure, track, and record the complete motion trajectory and configuration of the origami spring structure (shown in Fig. 5a). By capturing the movement of the structure, valuable data is obtained for further analysis; The control system, on the other hand, consists of servo motors and custom-designed 3D-printed mounting devices (shown in Fig. 5b, c). These servo motors serve as the actuators, responsible for controlling the folding angles of the origami spring structure. Depending on the number of independent variables, the control system can provide up to 4 actuators, offering flexibility in shape customization; To ensure the origami spring structure’s rigidity during operation, specific materials are utilized. The facets of the structure are constructed using 3D-printed polylactic acid (PLA), while the creases are made of polyethylene film (as depicted in Fig. 5b, d) that are thin (0.02 mm) and light, and have a high tensile modulus that ensures minimal stretching deformation during folding, allowing the facets to rotate about the creases as intended. The above material selections and fabrication processes provide the necessary stability and flexibility for the folding motion of the origami spring structure. In addition, to minimize the effects of facet thickness on folding, notches were reserved along the edges where facets are connected. These notches could prevent undesired contact during the folding process. To facilitate accurate tracking and identification by the motion capture system, markers are mounted on the extension of the origami spring structure. These target points act as markers, aiding the optical motion capture system in precisely capturing the structure’s movements and configurations. By integrating these components and materials, the experimental setup enables the comprehensive investigation of the origami spring structure’s behavior, including its folding motion, control mechanism, and accurate data acquisition.

**Fig. 6:**
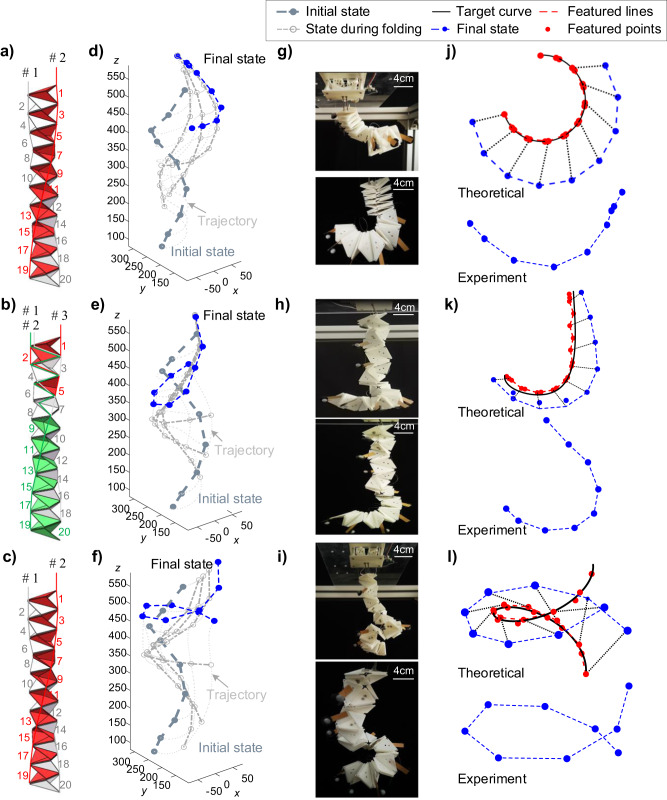
Experimental verification of using origami spring structure to approximate a ‘C’-shaped curve, a ‘J’-shaped curve, and a spiral-shaped curve. **a**–**c** Schematically illustrate the wiring method; the cells with the same actuation angles are marked with the same color, and detailed actuation angles are listed in Supplementary Tables 1–3. **d**–**f** Show the measured trajectory (dotted gray lines) and configurations (dashed gray lines) during actuation. **g**–**i** Show the front and side view of the final configuration (dashed blue lines). **j**–**l** Show the theoretical and experimental results for the final configuration.

In this experimental verification, we re-examine the ‘C’-shaped, ‘J’-shaped, and spiral-shaped curves as the target curves. The actuation angles for achieving these configurations with limited actuators can be found in Supplementary Tables 1–3. Note that a subset of unit cells shares identical actuation angles. This allows these unit cells to be driven collectively by a single actuator. Specifically, we threaded the actuation cable sequentially through these unit cells with the same actuation angles, which are depicted with the same color in Fig. 6a–c. This threading strategy enables synchronized folding of these cells, resulting in coordinated deformations. Based on the optimization results shown in Fig. 4, two independent actuators suffice the approximation of ‘C’ and spiral-shaped curves due to their inherent deformation modes, while the ‘J’-shaped curve demands three independent actuators to ensure accurate approximation, as its intricate geometry necessitates a more complex actuation scheme. The entire structure is driven by the servo motors fixed to the top acrylic plate. The actuation length refers to the amount of shortening of the actuation cable when folding the origami spring structure. Note that the actuation cable is arranged between vertices ${A}_{i}$ and ${C}_{i}$ of each unit cell, the actuation length can then be calculated from the change in distance between these two vertices, denoted as $\varDelta \Vert {A}_{i}{C}_{i}\Vert$. Depending on the number of unit cells (denoted as ${M}_{k}$, $k=1,2,\cdots ,m$, see Supplementary Tables 1–3) sharing the same actuation angle, the total actuation length can be expressed as ${M}_{k}\varDelta \Vert {A}_{i}{C}_{i}\Vert$.

During the experiment, the servo motors are driven synchronously to actuate the origami spring structure (detailed video recordings can be found in Supplementary Video). The configurations and trajectory of the structure are recorded using the Vicon optical motion capture system, as shown in Fig. 6d–f. The captured data provides valuable insights into the behavior and motion of the origami spring structure throughout the actuation process. After the actuation, the final state of the origami spring structure is visualized from the front and side views in Fig. 6g–i. In order to compare with the experimental results, we also calculated the theoretical spatial location of the markers on the extension lines. The obtained curvature closely resembles the experimental measurement, as shown in Fig. 6j–l, quantitative comparisons are available in Supplementary Discussion 2. This indicates the effectiveness of the optimization method in approximating the desired shapes. However, it is important to note that there may be slight disparities between the achieved configuration and the target curve, which primarily arise from the actuation errors of the individual unit cells. Specifically, cells near the drive end of the structure tend to have smaller angles compared to the theoretical values because of the influence of gravity and friction. Despite these small variations, the overall shape of the origami spring structure remains highly consistent with the desired target curve. The analysis of the experimental results provides valuable insights into the limitations and sources of error in the shape customization process. By understanding these factors, further improvements and refinements can be made to enhance the accuracy and reliability of the origami spring structure in approximating complex 3D curves.

While our current experiments focused on rigid folding origami structures with virtual creases, we recognize their limitations in representing the flexibility of origami spring structures typically made from flexible materials. Our future research direction will involve working with flexible materials, necessitating the development of more accurate flexible models and advanced optimization methods. This approach will allow us to explore the intricacies of flexible origami structures in a more precise manner, aligning our work more closely with real-world applications.

## Conclusion

In conclusion, our study has explored the potential of origami spring structures as a solution for approximating complex 3D curves and achieving intricate configurations using a limited number of actuators. By introducing virtual creases and incorporating rotation and translation transformations, we have established the spatial kinematics of origami springs, allowing for accurate descriptions of their spatial configurations. The development of inverse kinematics optimization algorithms has further enabled the determination of optimal configurations that closely approximate desired 3D curves, facilitating the creation of diverse and complex shapes. Moreover, our research has addressed the challenges associated with underactuated situations, unlocking possibilities in reconfigurable origami structures, soft robotics, flexible mechanisms, and morphing structures. To validate our approach, we have conducted a series of experimental verifications, providing tangible evidence of the feasibility and effectiveness of our proposed method.

As highlighted in this paper, the application of an origami spring structure to approximate the shape of a monkey tail is a prime example of the potential application of this structure in the field of bioinspired soft robotics. The origami spring structure could be used to develop soft robots emulating monkey tails, specialized in grasping elongated objects. The significance of our proposed methodology extends far beyond this special case. Given the inherent reconfigurability of the origami spring structure and its ability to accurately approximate diverse three-dimensional shapes under the proposed optimization-based approach, it opens the door to exploring and implementing a wide array of biomimetic structures. For instance, structures similar to an elephant trunk, a squid tentacle, or an inchworm body can be realized using the proposed method. These biomimetic structures have excellent adaptability to various grasping and locomotion scenarios, thereby providing unlimited possibilities for future soft robot development.

## Methods

### Geometry and kinematics of origami spring structures

The simplified unit cell is a one-degree-of-freedom system. Its folding can be uniquely characterized by folding angle ${\theta }_{i}$ (i.e., $\angle {D}_{i}{O}_{i}{B}_{i}$, ${O}_{i}$ lies in the middle of ${C}_{i}{E}_{i}$). Therefore, the coordinates of each vertex can be derived by

1 $$\left\{\begin{array}{c}\overrightarrow{{O}_{i}{A}_{i}}=-\overrightarrow{{O}_{i}{C}_{i}},\hfill\\ \overrightarrow{{O}_{i}{D}_{i}}=\overrightarrow{{O}_{i}{B}_{i}}\,\cos {\theta }_{i}+(\overrightarrow{{O}_{i}{B}_{i}}\times \overrightarrow{{A}_{i}{C}_{i}})\sin {\theta }_{i},\,\\ \overrightarrow{{C}_{i}{E}_{i}}=\overrightarrow{{C}_{i}{A}_{i}}\,\cos {\beta }_{i}+(\overrightarrow{{C}_{i}{A}_{i}}\times \overrightarrow{{D}_{i}{B}_{i}})\sin {\alpha }_{i},\end{array}\right.$$

where folding angle ${\alpha }_{i}$ (i.e., $\angle {A}_{i}{E}_{i}{C}_{i}$) is a function of ${\theta }_{i}$:

2 $${\alpha }_{i}=\arccos \left(\frac{1-\,\sin {\theta }_{i}}{3+\,\cos {\theta }_{i}}\right).$$

For an origami spring structure with n unit cells, the adjacent cells share one triangular plane (Fig. 1e), therefore, their coordinates follow

3 $${A}_{i+1}={C}_{i},{B}_{i+1}={D}_{i},{C}_{i+1}={E}_{i}.$$

Thus, the kinematics of the ${(i+1)}{{{{{{\rm{th}}}}}}}$ unit cell can be determined by the rotation and translation transformation. Here, ${O}_{i}$ and ${O}_{i+1}$ are chosen as origins, and the corresponding cartesian coordinates are shown in Fig. 1e. Based on the spatial relationship, the translation vector $\phantom{-}_{{O}_{i}}^{{O}_{i+1}}{\bf{P}}$ and rotation matrix $\phantom{-}_{{O}_{i}}^{{O}_{i+1}}{{\bf{R}}}$ of coordinate system $\{{O}_{i+1}\}$ with respect to coordinate system $\{{O}_{i}\}$ can be derived as follows

4 $$\begin{array}{c} \phantom{-}_{{O}_{i}}^{{O}_{i+1}}{{{{{\bf{P}}}}}}={\left[\begin{array}{cccc}{p}_{1} & {p}_{2} & {p}_{3} & 1\end{array}\right]}^{{\rm{T}}},\\ \phantom{-}_{{O}_{i}}^{{O}_{i+1}}{{\bf{R}}}=\left[\begin{array}{ccc} {r}_{11} & {r}_{12} & {r}_{13}\\ {r}_{21} & {r}_{22} & {r}_{23}\\ {r}_{31} & {r}_{32} & {r}_{33}\end{array}\right],\end{array}$$

where

5 $${p}_{1}= \,	a(\cos {\beta }_{i}-1),\,{p}_{2}=\frac{a\,\sin {\beta }_{i}\,\sin {\theta }_{i,j}}{\sqrt{2(1-\,\cos {\theta }_{i,j})}},\,{p}_{3}=a\,\sin {\beta }_{i}\sqrt{\frac{1-\,\cos {\theta }_{i,j}}{2}},\\ {r}_{11} = \,	-\,\cos {\beta }_{i},\,{r}_{12}=\frac{1-\,\cos {\beta }_{i}}{{Q}_{i}},\,{r}_{13}=-\frac{\sin {\beta }_{i}\sqrt{2(1-\,\cos {\theta }_{i,j})}}{{W}_{i}},\\ {r}_{21} = \,	-\frac{\sin {\beta }_{i}\,\sin {\theta }_{i,j}}{\sqrt{2(1-\,\cos {\theta }_{i,j})}},\,{r}_{22}=\frac{\cos {\theta }_{i,j}}{{Q}_{i}}-\frac{\sin {\beta }_{i}\,\sin {\theta }_{i,j}}{{Q}_{i}\sqrt{2(1-\,\cos {\theta }_{i,j})}},\,\\ {r}_{23} = \,	\frac{2\,\cos {\beta }_{i}\,\sin {\theta }_{i,j}-\sqrt{2(1-\,\cos {\theta }_{i,j})}\sin {\beta }_{i}}{{W}_{i}},\\ {r}_{31}= \,	-\frac{\sin {\beta }_{i}\sqrt{1-\,\cos {\theta }_{i,j}}}{\sqrt{2}},\,{r}_{32}=\,	\frac{\sqrt{2}\,\sin {\theta }_{i,j}-\,\sin {\beta }_{i}\sqrt{(1-\,\cos {\theta }_{i,j})}}{{Q}_{i}\sqrt{2}},\,\\ {r}_{33}= \,	\frac{\sqrt{2}\,\sin {\beta }_{i}\,\sin {\theta }_{i,j}}{{W}_{i}\sqrt{(1-\,\cos {\theta }_{i,j})}}-\frac{2\,\cos {\beta }_{i}\,\cos {\theta }_{i,j}}{{W}_{i}},\,$$

$$\,{Q}_{i}=\,	 \sqrt{3-2\,\cos {\beta }_{i}-\sqrt{2}\,\sin {\beta }_{i}\,\sin {\theta }_{i,j}/\sqrt{1-\,\cos {\theta }_{i,j}}},\\ {W}_{i} = \,	\sqrt{5-\,\cos {\theta }_{i,j}-\,\cos (2{\beta }_{i})(1-\,\cos {\theta }_{i,j})-2\sqrt{2}\,\sin (2{\beta }_{i})\sin {\theta }_{i,j}/\sqrt{1-\,\cos {\theta }_{i,j}}}.$$

From this, the coordinate transformation matrix of the adjacent cells yields

6 $$\phantom{-}_{{O}_{i}}^{{O}_{i+1}}{{\bf{T}}}={\left[\begin{array}{ll} \phantom{-}_{{O}_{i}}^{{O}_{i+1}}{\bf{R}} & \phantom{-}_{{O}_{i}}^{{O}_{i+1}}{\bf{P}}\\ {\bf{0}} & {\bf{1}}\end{array}\right]}_{4\times 4}.$$

With the coordinate transformation matrix (6), any vertex defined in the coordinate system $\{{O}_{i}\}$ can be derived via their description in $\{{O}_{i+1}\}$ (denoted as $\phantom{-}^{{O}_{i+1}}{\bf{V}}$, $\phantom{-}^{{O}_{i+1}}{\bf{V}}\in {\mathbb{R}}^{4\times 1}$)

7 $$\phantom{-}^{{O}_{i}}{\bf{V}}=\phantom{-}^{{O}_{i+1}}_{{O}_{i}}{{\bf{T}}}^{{O}_{i+1}}{\bf{V}}.$$

Similarly, the vertex defined in the coordinate system $\{{O}_{1}\}$ can be written as

8 $$\phantom{-}^{{O}_{1}}{{\bf{V}}}= \phantom{.}_{{O}_{i}}^{{O}_{2}}{{\bf{T}}}\phantom{-}_{{O}_{i}}^{{O}_{3}}{\bf{T}}\cdots \phantom{.}_{{O}_{n-1}}^{{O}_{n}}{{\bf{T}}}\phantom{-}^{{O}_{n}}{{\bf{V}}}.$$

Using this approach, it becomes possible to determine the position of any vertex within the origami spring structure relative to the base coordinate system. Consequently, a wide range of deformation modes exhibited by the origami spring structure can be accurately described and analyzed using this methodology.

### Generalized algorithm for approximating 3D curves with full actuation

The overall distance is determined by the sum of the distances between the featured points and their corresponding feet of perpendicular on the 3D curve. It is important to note that the first and last points of the origami spring are fixed on the target 3D curve. Therefore, the overall distance yields

9 $$S=\mathop{\sum }\limits_{i=1}^{n}{s}_{i},\,{s}_{i}=\Vert {O}_{i}{P}_{i}\Vert \,.$$

The spatial configuration of the origami spring structure can therefore be obtained by solving the following optimization problem:

10 $$\mathop{\min }\limits_{{{{{{\boldsymbol{\theta }}}}}}} 	\quad S\,({\theta }_{1},{\theta }_{2},\cdots ,{\theta }_{n}),\\ {{{{{\rm{subject}}}}}} 	\quad {{{{{\rm{to}}}}}}\\ 	\quad \Vert {O}_{0}{P}_{0}\Vert =0,\Vert {O}_{n+1}{P}_{n+1}\Vert =0.$$

In this paper, the 3D curves are described by parametric equations

11 $$x=g(t),y=k(t),z=m(t),t\in [{t}_{\min },{t}_{\max }].$$

Therefore, the feet of perpendicular ${P}_{i}$ can be easily determined by

12 $$\mathop{\min }\limits_{{t}_{i}}{\left({x}_{{O}_{i}}-g({t}_{i})\right)}^{2}+{\left({y}_{{O}_{i}}-k({t}_{i})\right)}^{2}+{\left({z}_{{O}_{i}}-m({t}_{i})\right)}^{2},$$

and the optimization problem can be rewritten as

13 $$	\mathop{\min }\limits_{{{{{{\boldsymbol{\theta }}}}}},{{{{{\bf{t}}}}}}} S \,({\theta }_{1},{\theta }_{2},\cdots ,{\theta }_{n},{t}_{1},{t}_{2},\cdots ,{t}_{n}),\\ 	{{{{{\rm{subject}}}}}}\, {{{{{\rm{to}}}}}}\\ 	\quad\quad\quad \left\{\begin{array}{c}{\left({x}_{{O}_{1}}-g({t}_{\min })\right)}^{2}+{\left({y}_{{O}_{1}}-k({t}_{\min })\right)}^{2}+{\left({z}_{{O}_{1}}-m({t}_{\min })\right)}^{2}=0,\\ {\left({x}_{{O}_{n}}-g({t}_{\max })\right)}^{2}+{\left({y}_{{O}_{n}}-k({t}_{\max })\right)}^{2}+{\left({z}_{{O}_{n}}-m({t}_{\max })\right)}^{2}=0.\end{array}\right.$$

The optimization process is conducted using the *fmincon* optimizer in Matlab, which could successfully find the optimal solution.

### Generalized algorithm for approximating 3D curves with underactuation

In the scenario where there are $m$ ($m\le n$) independent cells and the control angles are denoted as ${\gamma }_{j}$ ($j=1,2,\cdots ,m$), each cell ${\theta }_{i}$ ($j=1,2,\cdots ,n$) could take one of the control angles. We introduce the Lagrange multiplier method. Specifically, the large number of possible combinations could be approximately satisfied by solving the following optimization problem:

14 $$	\mathop{\min }\limits_{{{{{{\boldsymbol{\theta }}}}}},{{{{{\bf{t}}}}}},{{{{{\boldsymbol{\gamma }}}}}}}S\,({\theta }_{1},{\theta }_{2},\cdots ,{\theta }_{n},{t}_{1},{t}_{2},\cdots ,{t}_{n})+\lambda \mathop{\sum }\limits_{i=1}^{n}{({\theta }_{i}-{\gamma }_{1})}^{2}{({\theta }_{i}-{\gamma }_{2})}^{2}\cdots {({\theta }_{i}-{\gamma }_{m})}^{2},\\ 	{{{{{\rm{subject}}}}}}\; {{{{{\rm{to}}}}}}\\ 	\quad\qquad \left\{\begin{array}{c}{\left({x}_{{O}_{1}}-g({t}_{\min })\right)}^{2}+{\left({y}_{{O}_{1}}-k({t}_{\min })\right)}^{2}+{\left({z}_{{O}_{1}}-m({t}_{\min })\right)}^{2}=0, \\ {\left({x}_{{O}_{n}}-g({t}_{\max })\right)}^{2}+{\left({y}_{{O}_{n}}-k({t}_{\max })\right)}^{2}+{\left({z}_{{O}_{n}}-m({t}_{\max })\right)}^{2}=0,\end{array}\right.$$

where $\lambda$ is the Lagrange multiplier which takes a large number in the optimization. Therefore, the cell angle ${\theta }_{i}$ would atomically approximate one of the control angles in order to minimize the overall loss. This process aims to find an initial approximation of the cell angles, which is defined as the first optimization.

After the first optimization, although the cell angles are very close to the control angles, there may still be small differences between them. To further address this discrepancy, we proceed with a second optimization step. In this step, we directly set the close cell angles to be equal to the corresponding control angles. By doing so, we reduce the number of independent variables to be optimized. As a result, in the second optimization, there are only $m$ ($m\le n$) independent control angles that need to be optimized. The remaining cell angles have already been set to their corresponding control angles, eliminating the need for further adjustment:

15 $$	\mathop{\min }\limits_{{{{{{\bf{t}}}}}},{{{{{\boldsymbol{\gamma }}}}}}} \,\, S^{\prime} \,({\gamma }_{1},{\gamma }_{2},\cdots ,{\gamma }_{m},{t}_{1},{t}_{2},\cdots ,{t}_{n}),\\ 	{{{{{\rm{subject}}}}}} \,\, {{{{{\rm{to}}}}}}\\ 	\left\{\begin{array}{c}{\left({x}_{{O}_{1}}-g({t}_{\min })\right)}^{2}+{\left({y}_{{O}_{1}}-k({t}_{\min })\right)}^{2}+{\left({z}_{{O}_{1}}-m({t}_{\min })\right)}^{2}=0,\\ {\left({x}_{{O}_{n}}-g({t}_{\max })\right)}^{2}+{\left({y}_{{O}_{n}}-k({t}_{\max })\right)}^{2}+{\left({z}_{{O}_{n}}-m({t}_{\max })\right)}^{2}=0.\end{array}\right.$$

We define this process as the Second optimization. By conducting these two optimization steps, we are able to effectively approximate the desired shape while reducing the number of independent variables, which helps to streamline the optimization process and reduce computational complexity.

### Supplementary information

Supplementary Information

Description of Additional Supplementary Files

Supplementary Video

## Data Availability

The data that support the plots within this paper and other findings of this study are available from the author upon reasonable request

## Code availability

The code that supports the findings of this study is available from the corresponding author upon reasonable request.
