# Supplementary material for: Approximating complex 3D curves using origami spring structures
Source: Commun Eng. 2023 Dec 12;2:90. doi: 10.1038/s44172-023-00149-1 (PMC11101438; doi:10.1038/s44172-023-00149-1)
Supplement: Supplementary file 1 — Supplementary Information [file 44172_2023_149_MOESM1_ESM.pdf]

Supplementary Information for

Approximating complex 3D curves using origami spring structures

Zuolin Liu, Zian Zhang, and Hongbin Fang

Supplementary Notes: Parametric equation of the curves

Expressions for several 3D curves used in the main text are given in this section. Of these, the C-, spiral and J-shaped curves are given in parametric equations, the sinusoidal-shaped curve is given in the general form, and the monkey-tail type is given after fitting using parametric equations since there is no specific expression for it.

'C'-shaped curve：

$$\begin{cases} x = -57\sin t, \\ y = 4.4t, \\ z = 57 - 57\cos t, \end{cases} \qquad t \in (0, \pi). \tag{1}$$

Spiral-shaped curve：

$$\begin{cases} x = 45 - (45 + 0.8t)\cos t, \\ y = (-70 + 5t)\sin t, \\ z = -25t, \end{cases} \qquad t \in (0, 3/2\pi). \tag{2}$$

'J'-shaped curve：

$$\begin{cases} x = -0.2097t + 2.1756t^2 + 0.4219t^3 - 0.1696t^4 + 0.0139t^5 - 0.0003t^6, \\ y = 1.0856t - 3.1131t^2 + 1.5701t^3 - 0.2385t^4 + 0.0190t^5 - 0.0007t^6, \\ z = 23.2190t + 11.1638t^2 - 3.5821t^3 + 0.4288t^4 - 0.0248t^5 + 0.0006t^6, \end{cases} \qquad t \in (0, 10). \tag{3}$$

Sinusoidal-shaped curve (in general form)：

$$\begin{cases} x = 100\sin(-5/z), \\ y = -20z, \end{cases} \qquad z \in (-10\pi, 0). \tag{4}$$

Monkey tail-shaped curve (approximated with parametric equation)：

$$\begin{cases} x = -2.6656t + 5.1748t^2 - 5.1014t^3 + 0.9468t^4 - 0.0492t^5, \\ y = -1.1445t - 5.3801t^2 + 6.2472t^3 - 2.2777t^4 + 0.2889t^5 - 0.0119t^6, \\ z = 46.2160t + 5.0709t^2 - 1.1546t^3 + 0.0153t^4 + 0.0035t^5, \end{cases} \qquad t \in (0, 10). \tag{5}$$

Supplementary Discussion 1: Reachable workspace of the origami spring structure with the routing paths of tendons for 'C'-shaped curves

For a given threading method, such as the routing paths of tendons that approximate the ‘C’-shaped curve (see Supplementary Table 1), the corresponding reconfigurability of the origami spring structure is notably rich. Under this threading scheme, the origami structure possesses two degrees of freedom, and by systematically exploring the possible driving angles, we chart the reachable workspace, as depicted in Supplementary Figure 1. It becomes evident that the potential locations of its structural endpoints collectively form a complex spatial surface, highlighting the structure's extensive motion capability. In addition to presenting a ‘C’-shaped configuration, the spatial configuration can also manifest as a spiral (as illustrated by the blue line in the figure). Remarkably, this aligns perfectly with the optimization results for the spiral-shaped curve, which exhibits the same routing paths as the ‘C’-shaped curve.

It is crucial to note that within such routing paths, there exist infinite ranges of motion, indicating that a predefined routing path can yield a variety of distinct spatial configurations. This underscores the remarkable reconfigurability inherent in origami spring structures. These characteristics hold significant implications for the realization of versatile reconfigurable robots, enhancing their adaptability to various environments.

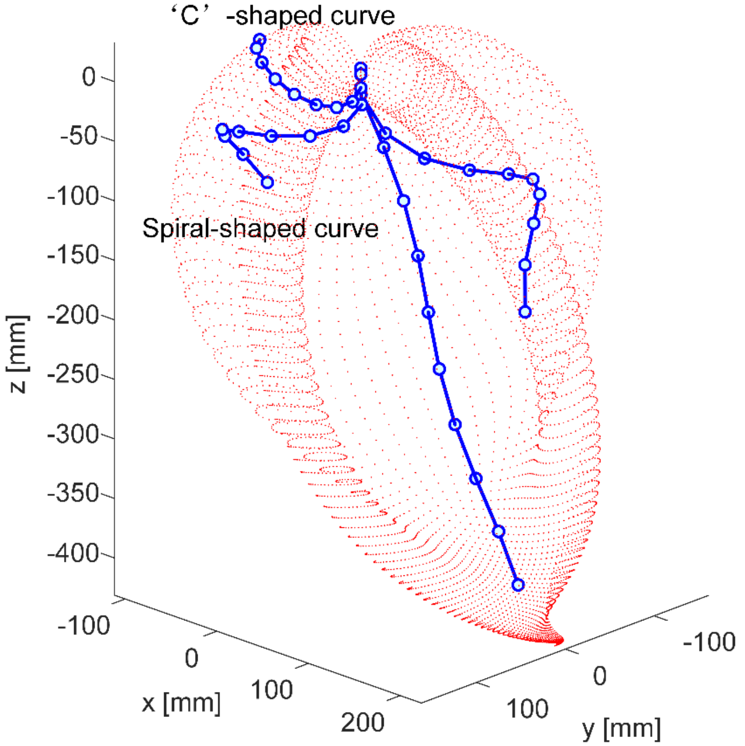

Supplementary Figure 1 Reachable workspace of the origami spring structure with the routing paths of tendons for ‘C’-shaped curves. The spatial configuration for ‘C’ and Spiral-shaped curves are denoted. Two other spatial configurations are also given.

Supplementary Discussion 2: Comparison of the experimental results with the theoretical prediction

In addition to the quantitative experimental verification presented in Figure 6 of the main text, this section provides quantitative results to further demonstrate the effectiveness of our proposed method. To facilitate a more precise comparison between the two sets of curves, we performed translations and rotations on the theoretical curves as a whole, while preserving their spatial shapes, to bring them into closer alignment with the experimental curves.

Overall, these three curves, namely the 'C'-, 'J'-, and spiral-shaped curves (as shown in Supplementary Figures 2-4, respectively), exhibit remarkably similar spatial patterns compared to their theoretical predictions. This similarity is particularly evident when examining their projections onto the coordinate axes, which closely coincide. This reinforces the validation of our optimization and actuation strategies.

However, due to the gravity and other factors, such as friction and uncertainties, there are still disparities between the experimental curves and the theoretical curves, especially in the context of 3D space. One avenue for improvement is the development of more sophisticated kinematic and dynamic models that account for these real-world factors. This will allow us to refine our theoretical predictions and bring them more consistent with experiments. Additionally, the introduction of closed-loop control strategies is another promising direction to further enhance control precision.

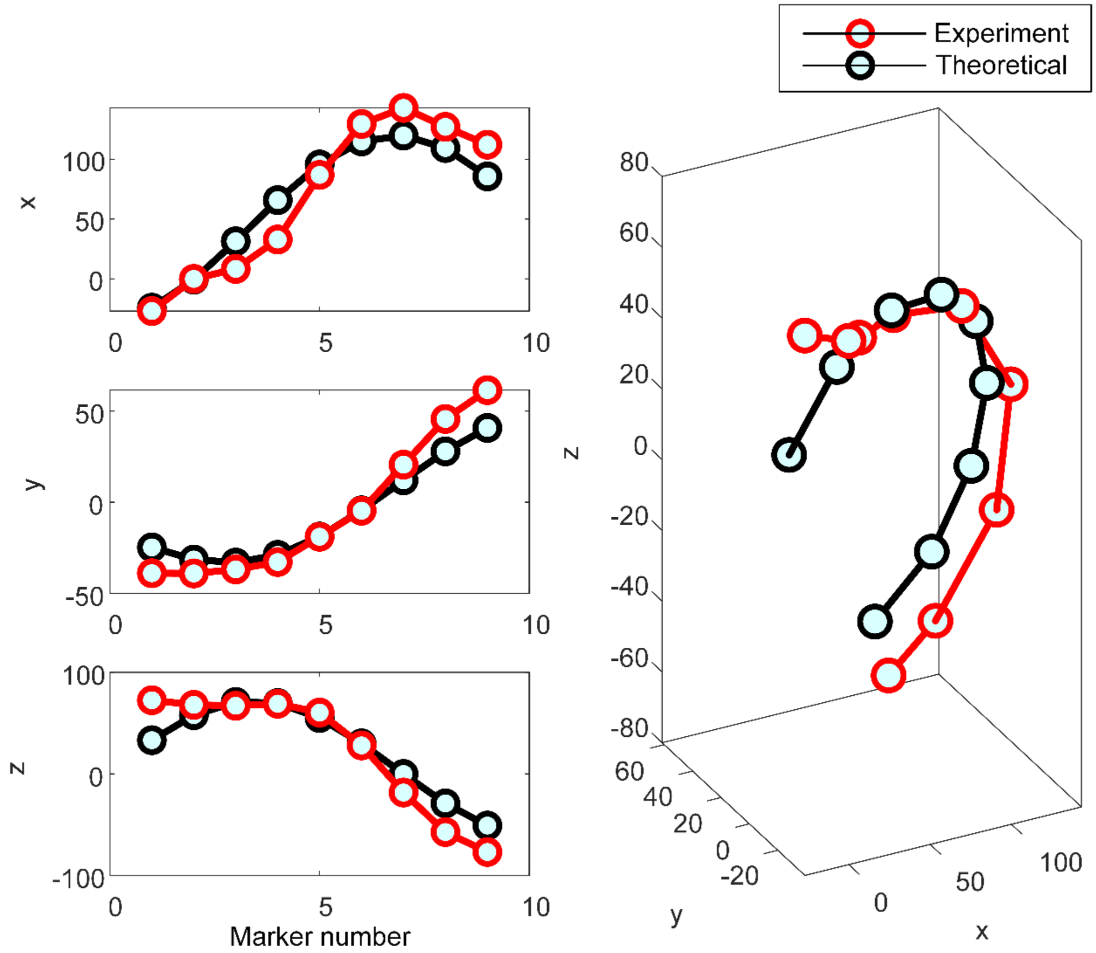

Supplementary Figure 2 Comparison between experimental results and theoretical predictions for approximating the 'C'-shaped curve. The left panels display the projections of the two 3D curves along the three axes, while the right panel illustrates their configurations in 3D space.

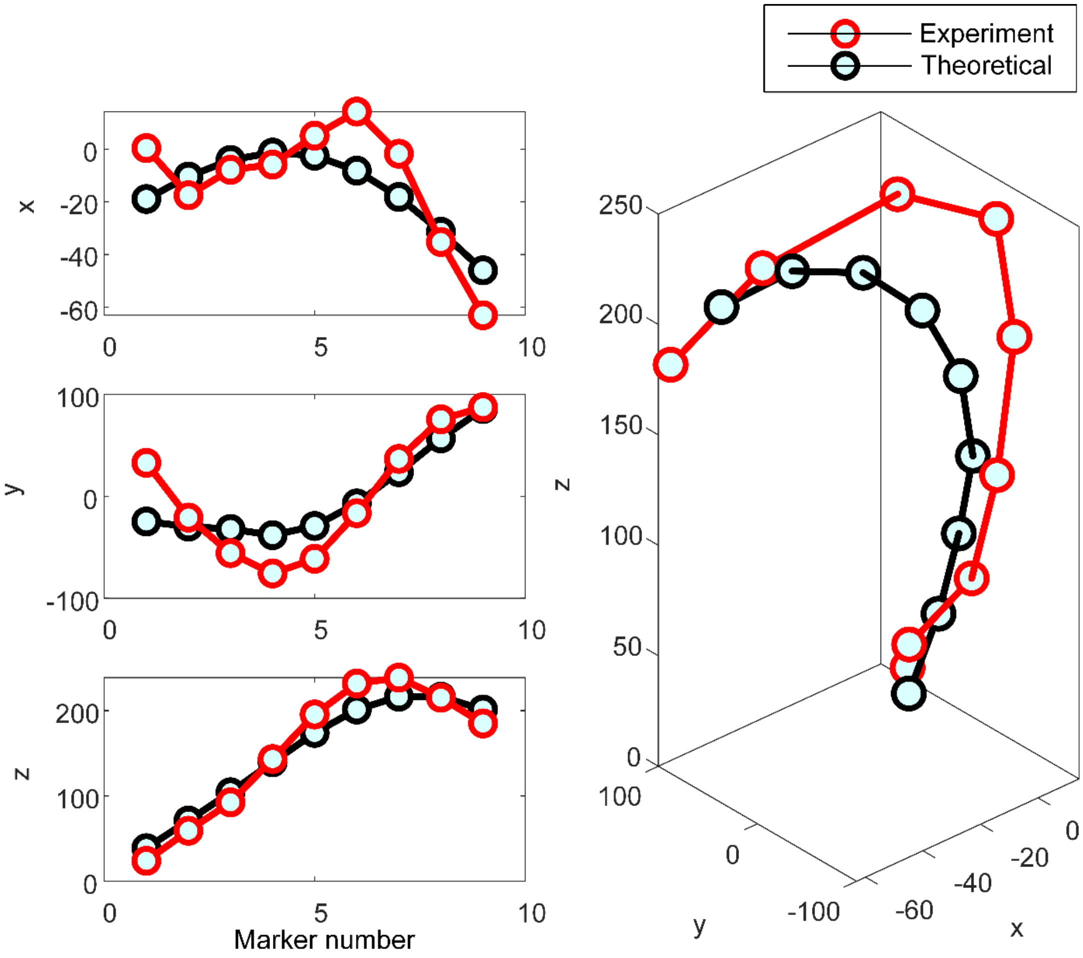

Supplementary Figure 3 Comparison between experimental results and theoretical predictions for approximating the 'J'-shaped curve. The left panels display the projections of the two 3D curves along the three axes, while the right panel illustrates their configurations in 3D space

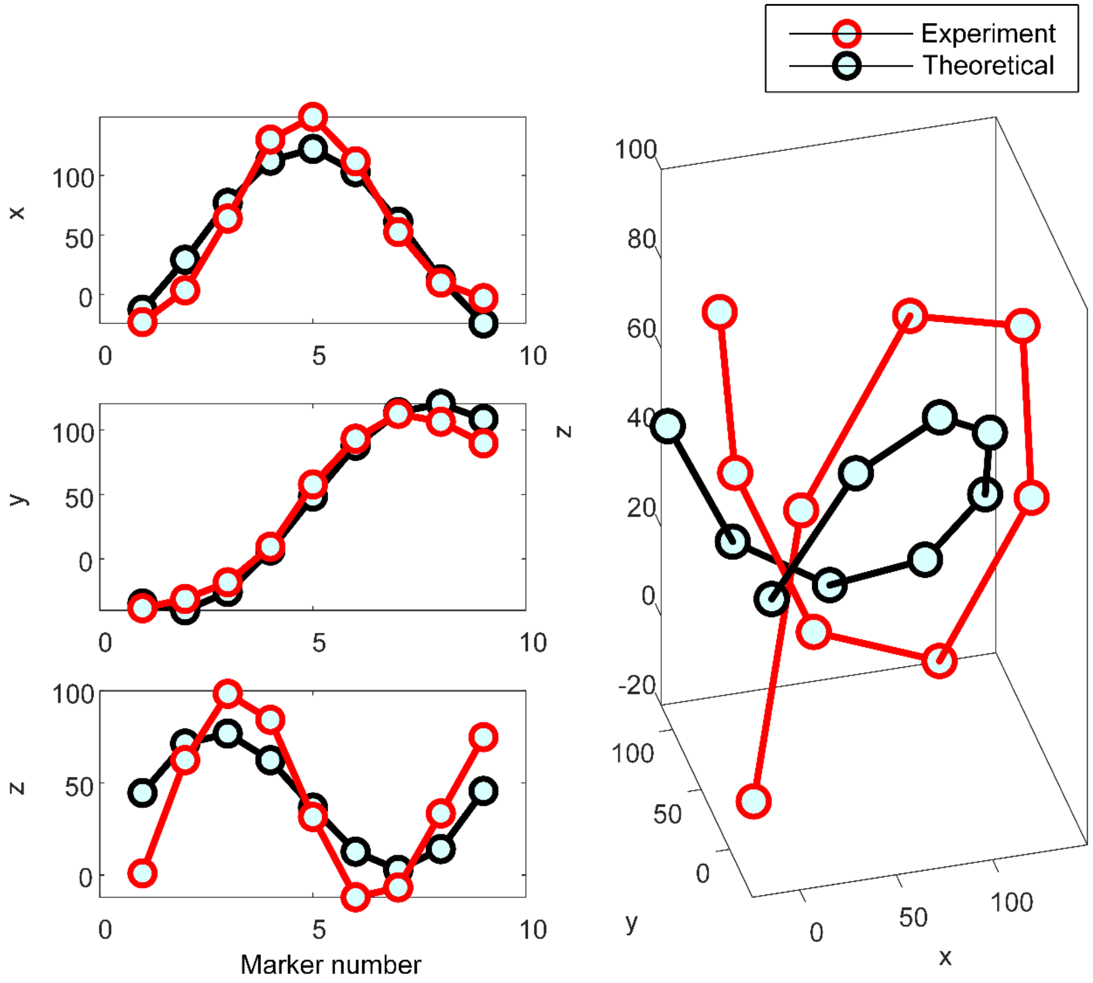

Supplementary Figure 4 Comparison between experimental results and theoretical predictions for approximating the spiral-shaped curve. The left panels display the projections of the two 3D curves along the three axes, while the right panel illustrates their configurations in 3D space

Supplementary Table 1 actuation angles for 'C'-shaped curve after the first and the second optimizations

Index	1	3	5	7	9	11	13	15	17	19
Angle(1st)	172.35	172.37	172.35	172.28	172.34	172.34	172.37	172.38	172.36	172.33
Angle(2ed)	172.38									
Index	2	4	6	8	10	12	14	16	18	20
Angle(1st)	145.79	145.79	145.81	145.78	145.79	145.86	145.79	145.80	145.80	145.78
Angle(2ed)	145.82									

Supplementary Table 2 actuation angles for 'J'-shaped curve after the first and the second optimizations

Index	1	2	5	9	11	13	15	17	19	20
Angle(1st)	159.93	154.91	152.95	166.64	167.54	167.97	169.27	167.65	166.96	168.40
Angle(2ed)		152.85					168.72			
Index	3	4	6	7	8	10	12	14	16	18
Angle(1st)	134.97	134.87	135.85	135.58	135.79	135.43	135.57	135.25	136.04	135.72
Angle(2ed)						137.61				

Supplementary Table 3 actuation angles for spiral-shaped curve after the first and the second optimizations

Index	1	3	5	7	9	11	13	15	17	19
Angle(1st)	162.92	162.92	162.73	162.34	162.53	162.53	162.25	161.93	162.16	162.30
Angle(2ed)						162.40				
Index	2	4	6	8	10	12	14	16	18	20
Angle(1st)	125.56	126.86	126.77	126.04	126.69	125.20	126.59	126.76	126.37	126.66
Angle(2ed)					126.61					

Supplementary Table 4 Relative error for various 3D curve after the optimizations

	'C'	'J'	Spiral	Sinusoidal	Monkey tail
Full actuation (Fig. 2)	0.24%	0.60%	0.66%	0.60%	1.81%
Underactuated (Fig. 4)	0.32%	0.91%	1.63%	-	-
