# Supplementary material for: Approximating complex 3D curves using origami spring structures
Source: Commun Eng. 2023 Dec 12;2:90. doi: 10.1038/s44172-023-00149-1 (PMC11101438; doi:10.1038/s44172-023-00149-1)
Supplement: Supplementary file 2 — Description of Additional Supplementary Files [file 44172_2023_149_MOESM2_ESM.pdf]

Description of Additional Supplementary Files

File name: Supplementary Video

Description: Experimental Demonstration of Using Origami Spring Structure to Approximate ‘C’-Shaped, ‘J’-Shaped, and Spiral-Shaped Curves.
Experimental demonstration of using origami spring structure to approximate ‘C’-shaped, ‘J’-shaped, and spiral-shaped curves in Figure 6. All three videos were recorded from a frontal perspective.
